# Diseases of the Thyroid in the Female

**Published:** 1912-02

**Authors:** 


Diseases of the Thyroid in the Female.—Miles F. Porter of Fort Wayne, Ind. (*Am. Jour. Obstet.* November 1911), is convinced that the thyroid gland deserves more consideration at the hands of gynaecologists and obstetricians than it has received. There is much clinical evidence in support of the theory that what may be termed a physiologic superactivity of the thyroid is a valuable safeguard against puerperal toxaemias and infections. He reports six cases which show the need of attention to the thyroid in disturbance of the sexual organs of women. His observation leads him to believe that those pale, poorly nourished, nervous young women who complain of some menstrual disorder or pelvic discomfort are more often the subjects of thyroid than of genital disease.

